# Supplementary material for: Determinants of Outdoor Time in Children and Youth: A Systematic Review of Longitudinal and Intervention Studies
Source: Int J Environ Res Public Health. 2023 Jan 11;20(2):1328. doi: 10.3390/ijerph20021328 (PMC9859594; doi:10.3390/ijerph20021328)
Supplement: Supplementary file 1 [file ijerph-20-01328-s001.zip › Supplemental Information 2 Systematic Review Search Terms.pdf]

Supplementary Material 2: search strategies for all databases, except MEDLINE[1]

**Figure S1 APA PsycINFO Search Strategy for Systematic Review**	
1	(boy* or girl* or child* or toddler* or adolescen* or juvenile* or teen or teens or teenage* or youth or youths).tw.
2	(young adj 1 (people or person*)).tw.
3	((primary or elementary or high or secondary) adj1 school*).tw.
4	(schoolchild* or school child* or schoolage or highschool* or daycare or preschool*).tw.
5	or/1-4
6	exp recreation areas/
7	((outdoor* or outside or park*) adj5 (play* or time or learn* or educat*)).tw.
8	or/6-7
9	(randomized or randomised).ab,ti.
10	randomly.ab,ti.
11	trial.ab,ti.
12	exp Program Evaluation/
13	(cohort* or prospectiv* or observ* or longitudinal* or quasi-longitudinal or (case* and control*)).tw.
14	or/9-13
15	5 and 8 and 14
16	limit 15 to (english or french or japanese or spanish)
	Note: exp = used with a term to include all narrower terms; .ab, .ti, .pt, .tw = field codes for abstract, title, publication type, and text word, respectively; adj# = search for records with terms within # words of each other; * after keyword indicates truncation (e.g., adolescen* will retrieve “adolescent”, “adolescents”, “adolescence”, etc.)

[1] Note that the MEDLINE strategy is provided in Figure 1 (instead of the supplementary file).

Figure S2 CENTRAL Search Strategy for Systematic Review

1	exp Child/
2	exp Adolescent/
3	exp Infant/
4	(boy* or girl* or child* or toddler* or adolescen* or juvenile* or teen or teens or teenage* or youth or youths).tw.
5	(young adj1 (people or person*)).tw.
6	((primary or elementary or high or secondary) adj1 school*).tw.
7	(schoolchild* or school child* or schoolage or highschool* or daycare or preschool*).tw.
8	or/1-7
9	exp Parks, Recreational/
10	((outdoor* or outside or park*) adj5 (play* or time or learn* or educat*)).tw.
11	or/9-10
12	randomized controlled trial.pt.
13	controlled clinical trial.pt.
14	(randomized or randomised).ab,ti.
15	randomly.ab,ti.
16	trial.ab,ti.
17	exp Case-Control Studies/
18	exp Cohort Studies/
19	exp Program Evaluation/
20	(cohort* or prospectiv* or observ* or longitudinal* or quasi-longitudinal or (case* and control*)).tw.
21	or/12-20
22	8 and 11 and 21
23	limit 22 to (english or french or japanese or spanish)
	Note: exp = used with a term to include all narrower terms; .ab, .ti, .pt, .tw = field codes for abstract, title, publication type, and text word, respectively; adj# = search for records with terms within # words of each other; * after keyword indicates truncation (e.g., adolescen* will retrieve “adolescent”, “adolescents”, “adolescence”, etc.)

**Figure S3 CINAHL Search Strategy for Systematic Review**	
S20	S7 AND S8 AND S18
S19	S7 AND S8 AND S18
S18	S9 OR S10 OR S11 OR S12 OR S13 OR S14 OR S15 OR S16 OR S17
S17	(cohort* or prospectiv* or observ* or longitudinal* or quasi-longitudinal or (case* and control*))
S16	(MH “Program Evaluation”)
S15	(MH “Prospective Studies+”)
S14	(MH “Case Control Studies+”)
S13	(trial)
S12	(randomly)
S11	(randomized or randomised)
S10	(MH “Clinical Trials+”)
S9	(MH “Randomized Controlled Trials+”)
S8	((outdoor* or outside or park*) N5 (play* or time or learn* or educat*))
S7	S1 OR S2 OR S3 OR S4 OR S5 OR S6
S6	(schoolchild* or school child* or schoolage or highschool* or daycare or preschool*)
S5	((primary or elementary or high or secondary) N1 school*)
S4	(young N1 (people or person*))
S3	(boy* or girl* or child* or toddler* or adolescen* or juvenile* or teen or teens or teenage* or youth or youths)
S2	(MH “Adolescence+”)
S1	(MH “Child+”)
	Note: MH: searches both major and minor headings; N# = search for records with terms within # words of each other; * after keyword indicates truncation (e.g., adolescen* will retrieve “adolescent”, “adolescents”, “adolescence”, etc.)

Figure S4 ERIC Search Strategy for Systematic Review

S20	S8 AND S11 AND S18
S19	S8 AND S11 AND S18
S18	S12 OR S13 OR S14 OR S15 OR S16 OR S17
S17	(cohort* or prospectiv* or observ* or longitudinal* or quasi-longitudinal or (case* and control*)
S16	DE “Program Evaluation”
S15	trial
S14	randomly
S13	(randomized or randomized)
S12	DE “Randomized Controlled Trials”
S11	S9 OR S10
S10	((outdoor* or outside or park*) N5 (play* or time or learn* or educat*))
S9	DE “Parks”
S8	S1 OR S2 OR S3 OR S4 OR S5 OR S6 OR S7
S7	(schoolchild* or school child* or schoolage or highschool* or daycare or preschool*)
S6	((primary or elementary or high or secondary) N1 school*)
S5	(young N1 (people or person*))
S4	(boy* or girl* or child* or toddler* or adolescen* or juvenile* or teen or teens or teenage* or youth or youths)
S3	DE “Adolescents”
S2	DE “Infants” OR DE “Neonates” or DE “Premature Infants”
S1	DE “Children” OR DE “African American Children” OR DE “Grandchildren” OR DE “Hospitalized Children” OR DE “Latchkey Children” OR DE “Migrant Children” OR DE “Minority Group Children” OR DE “Preadolescents” OR DE “Young Children”

Note: DE: searches exact subject heading descriptors; N# = search for records with terms within # words of each other; * after keyword indicates truncation (e.g., adolescen* will retrieve “adolescent”, “adolescents”, “adolescence”, etc.)

**Figure S5 ProQuest Dissertations & Theses Search Strategy for Systematic Review**	
S13	S5 and S6 and S11
S12	S5 and S6 and S11
S11	S7 or S8 or S9 or S10
S10	noft ((cohort* or prospectiv* or observ* or longitudinal* or quasi-longitudinal or (case* and control*)))
S9	noft ((trial))
S8	noft((randomly))
S7	noft ((randomized or randomised))
S6	noft(((outdoor* or outside or park*) N/5 (play* or time or learn* or educat*)))
S5	S1 or S2 or S3 or S4
S4	noft((schoolchild* or school child* or schoolage or highschool* or daycare or preschool*))
S3	noft(((primary or elementary or high or secondary) N/1 school*))
S2	noft((young N/1(people or person*)))
S1	noft(boy* or girl* or child* or toddler* or adolescen* or juvenile* or teen or teens or teenage* or youth or youths)
	Note: noft = search for the keyword in all fields in the full record, except the full text; N/# = search for records with terms within # words of each other; * after keyword indicates truncation (e.g., adolescen* will retrieve “adolescent”, “adolescents”, “adolescence”, etc.)

Figure S6 SocINDEX Search Strategy for Systematic Review	
S22	S8 AND S11 AND S20
S21	S8 AND S11 AND S20
S20	S12 OR S13 OR S14 OR S15 OR S16 OR S17 OR S18 OR S19
S19	cohort* or prospectiv* or observ* or longitudinal* or quasi-longitudinal or (case* and control*)program evaluation
S18	DE "COHORT analysis"
S17	DE "CASE-control method"
S16	trial
S15	randomly
S14	randomized or randomised
S13	DE "CLINICAL trials"
S12	DE "RANDOMIZED controlled trials"
S11	S9 OR S10
S10	(outdoor* or outside or park*) N5 (play* or time or learn* or educat*)
S9	DE "PARKS" OR DE "NATIONAL parks & reserves" OR DE "URBAN parks"
S8	S1 OR S2 OR S3 OR S4 OR S5 OR S6 OR S7
S7	(schoolchild* or school child* or schoolage or highschool* or daycare or preschool*)
S6	(primary or elementary or high or secondary) N1 school*
S5	young N1 (people or person*)
S4	boy* or girl* or child* or toddler* or adolescen* or juvenile* or teen or teens or teenage* or youth or youths
S3	DE "TEENAGERS" OR DE "ABUSED teenagers" OR DE "BLACK teenagers" OR DE "CELL phones & teenagers" OR DE "CHRISTIAN teenagers" OR DE "GIFTED teenagers" OR DE "HOMELESS teenagers" OR DE "INTERNET & teenagers" OR DE "JEWISH teenagers" OR DE "LGBTQ+ teenagers" OR DE "MASS media & teenagers" OR DE "MENTALLY ill teenagers" OR DE "MINORITY teenagers" OR DE "MOTION pictures & teenagers" OR DE "MUSIC & teenagers" OR DE " MUSLIM teenagers" OR DE "OVERWEIGHT teenagers" OR DE "POLICE services for juvenile" OR
S2	DE "INFANTS" OR DE "INFANT boys" OR DE "INFANT girls" OR DE "INFANTS with disabilities" OR DE "NEWBORN Infants" OR DE "PREMATURE infants"
S1	DE "CHILDREN" OR DE "ABUSED children" OR DE "ADOPTED children" OR DE "ADULT children" OR DE "ADULT children of alcoholics" OR DE " ADVERTISING & children" OR DE "AIDS & children" OR DE "ARTS & children" OR DE "BAHAI children" OR DE "BILINGUALISM in children" OR DE "BIRTH order" OR DE "BLACK children" OR DE "BOYS" OR DE "BUDDHIST children" OR DE "CHILD beauty pageant contestants" OR DE "CHILD development" OR DE "CHILD patients" OR DE "CHILD prostitutes" OR DE "CHILDREN & erotica" OR DE " CHILDREN &…
	Note: DE: searches exact subject heading descriptors; N# = search for records with terms within # words of each other; * after keyword indicates truncation (e.g., adolescen* will retrieve "adolescent", "adolescents", "adolescence", etc.)

Figure S7 SPORTDiscus Search Strategy for Systematic Review

S20	S7 AND S10 AND S18
S19	S7 AND S10 AND S18
S18	S11 OR S12 OR S13 OR S14 OR S15 OR S16 OR S17
S17	(cohort* or prospectiv* or observ* or longitudinal* or quasi-longitudinal or (case* and control*))
S16	DE “COHORT analysis”
S15	DE “CASE-control method”
S14	(trial)
S13	(randomly)
S12	(randomized or randomised)
S11	DE “RANDOMIZED controlled trials”
S10	S8 OR S9
S9	((outdoor* or outside or park*) N5 (play* or time or learn* or educat*))
S8	DE “PARKS” OR DE “AQUATIC parks & reserves” OR DE “NATIONAL parks & reserves” OR DE “SKATEBOARDING parks” OR DE “PLAYGROUNDS”
S7	SI OR S2 OR S3 OR S4 OR S5 OR S6
S6	(schoolchild* or school child* or schoolage or highschool* or daycare or preschool*)
S5	((primary or elementary or high or secondary) N1 school*)
S4	(young N1 (people or person*))
S3	boy* or girl* or child* or toddler* or adolescen* or juvenile* or teen or teens or teenage* or youth or youths)
S2	DE “TEENAGERS” OR DE “ OVERWEIGHT teenagers” OR DE “VIDEO games & teenagers”
S1	DE “CHILDREN” OR DE “AIDS & children” OR DE “BOYS” OR DE “CHILD acrobats” OR DE “CHILD circus performers” OR DE “CHILD dancers” OR THE “CHILD development” OR DE “DANCE for children” OR DE “DEAFBLIND children” OR DE “GIRLS” OR DE “OUTDOOR recreation for children” OR DE “OVERWEIGHT children” OR DE “SCHOOL children” OR DE “SELF-defense for children” OR DE “VIDEO games & children”
	Note: DE: searches exact subject heading descriptors; N# = search for records with terms within # words of each other; * after keyword indicates truncation (e.g., adolescen* will retrieve “adolescent”, “adolescents”, “adolescence”, etc.)

Figure S8 Web of Science Search Strategy for Systematic Review

S13	(#12) AND LANGUAGE: (English OR French OR Japanese OR Spanish)
S12	#11 AND #6 AND #5
S11	#10 OR #9 OR #8 OR #7
S10	TS=(cohort* or prospectiv*or observ* or longitudinal* or quasi-longitudinal or (case* and control*))
S9	TS=(trial)
S8	TS=(randomly)
S7	TS=(randomized or randomised)
S6	TS=((outdoor* or outside or park*) NEAR/5 (play* or time or learn* or educat*))
S5	#4 OR #3 OR #2 OR #1
S4	TS=(schoolchild* or school child* or schoolage or highschool* or daycare or preschool*)
S3	TS=(primary or elementary or high or secondary) NEAR/1 school*)
S2	TS=(young NEAR/1(people or person*))
S1	TS=(boy* or girls* or child* or toddler* or adolescen* or juvenile* or teen or teens or teenage* or youth or youths)
	Note: TS = search for topic in the following fields: title, abstract, author keywords, and Keywords Plus®; NEAR/# = search for records with terms within # words of each other; * after keyword indicates truncation (e.g., adolescen* will retrieve "adolescent", "adolescents", "adolescence", etc.)
